# Supplementary material for: Interaction of surface cations of cleaved mica with water in vapor and liquid forms
Source: Faraday Discuss. 2023 Jun 9;249:84–97. doi: 10.1039/d3fd00093a (PMC10845011; doi:10.1039/d3fd00093a)
Supplement: FD-249-D3FD00093A-s001 [file FD-249-D3FD00093A-s001.pdf]

Electronic Supplementary Material (ESI) for Faraday Discussions.
This journal is © The Royal Society of Chemistry 2023

Supplementary Material –
Interaction of surface cations of cleaved mica with water in vapor and liquid forms

Giada Franceschi, Sebastian Brandstetter, Jan Balajka, Igor Sokolović, Jiri Pavelec, Martin Setvín, Michael Schmid, and Ulrike Diebold

S1. Water adsorption at room temperature

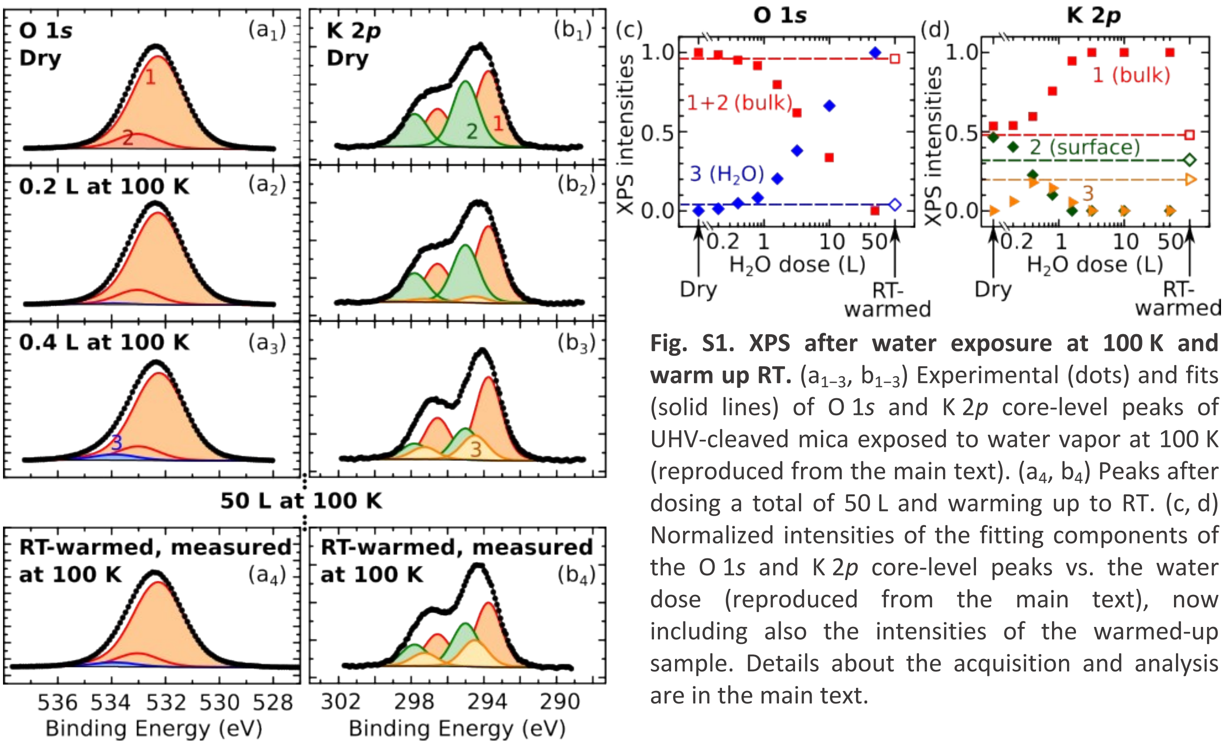

Fig. S1. XPS after water exposure at 100 K and warm up RT. (a_{1-3}, b_{1-3}) Experimental (dots) and fits (solid lines) of O 1*s* and K 2*p* core-level peaks of UHV-cleaved mica exposed to water vapor at 100 K (reproduced from the main text). (a_4, b_4) Peaks after dosing a total of 50 L and warming up to RT. (c, d) Normalized intensities of the fitting components of the O 1*s* and K 2*p* core-level peaks vs. the water dose (reproduced from the main text), now including also the intensities of the warmed-up sample. Details about the acquisition and analysis are in the main text.

Water does not adsorb at room temperature (RT) on the UHV-cleaved mica. Lowering the sample temperature increases the sticking. However, some water species remain after dosing water vapor at low temperature and warming up to RT, suggesting that the adsorption of water at room temperature is kinetically limited. Figure S1a_4,b_4 show the K 2*p* and O 1*s* peaks of a sample exposed to 50 L H_2O at 100 K, then warmed up to RT to let the water desorb, and cooled down again for acquiring XPS. (Figs. S1a_{1-3},b_{1-3} correspond to small coverages of water dosed at 100 K; they are reproduced from Fig. 3 of the main text). The peak shape is different from the one of the dry mica, an indication that some water species are still present on the surface (this is consistent with the appearance of nc-AFM images, which do not resemble the as-cleaved surface; not shown). Figures S1c,d include the quantitative analysis of the fitting components of the spectra of the warmed up sample (empty symbols). The values correspond to the ones that can be obtained by dosing between 0.2 L and 0.4 L water at 100 K on UHV-cleaved samples. This indicates that residual water is present on the sample's surface after warming up to RT. Note that acquiring XPS data at room temperature induces a change in the peak shape towards the "dry" spectrum – a sign of X-ray-stimulated desorption occurring at room temperature, possibly due to X-ray-induced electronic transitions.[1] It cannot be excluded that the residual water after warming up to RT is dissociated. Thermally activated dissociation was previously observed on $Fe_3O_4(001)$ at around 150 K.[2]

S2. Unusual tip conditions to measure surfaces exposed to water vapor at 100 K

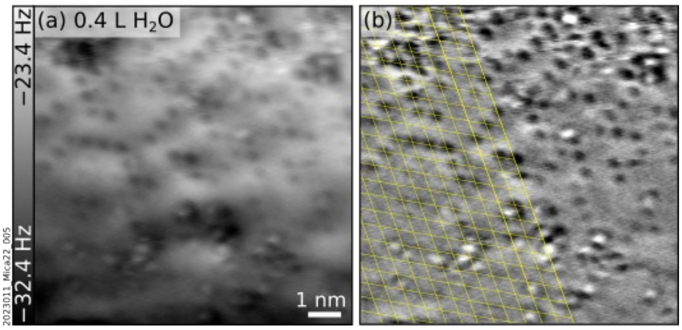

Fig. S2. (a, b) 9.6 × 9.6 nm^2 nc-AFM image of 0.4 L H_2O dosed at 100 K on a UHV-cleaved mica surface, acquired with an ill-defined tip. (b) Corresponding high-pass-filtered image; the yellow overlay highlights the unit cells of cleaved mica.

Figures S2a shows a nc-AFM image of the surface of a UHV-cleaved mica sample after exposure to 0.4 L H_2O at 100 K (the same coverage as Fig. 4b in the main text) acquired with an ill-defined tip – i.e., unintentionally modified after interaction with the surface. Similarly to the surface of dry mica,[3] one can see strong background modulations (this sample was measured at a bias voltage not compensating the LCPD) and resolve black (attractive) dots corresponding to the K^+ lattice of cleaved mica. In addition, bright (repulsive) features are visible in the lattice positions “between” the ion sites. The attractive and repulsive features are more evident in the high-pass-filtered image of Figure S2b.

Following the reasonings of the main text, the dark dots are assigned to K^+ ions unaffected by the small dose of water vapor introduced in the chamber. With standard tip preparations (Cu, CO-terminated), it was not possible to obtain atomic resolution on the K^+ lattice after dosing water vapor on the cleaved surface.

The authors refrain from further analysis of these images since the contrast obtained with this tip was rare.

References

1 R. A. Baragiola and T. E. Madey, in *Interaction of Charged Particles with Solids and Surfaces*, 1991, pp. 313–344.

2 M. Meier, J. Hulva, Z. Jakub, J. Pavelec, M. Setvin, R. Bliem, M. Schmid, U. Diebold, C. Franchini and G. S. Parkinson, *Proc. Natl. Acad. Sci. U. S. A.*, 2018, **115**, E5642–E5650.

3 G. Franceschi, P. Kocán, A. Conti, S. Brandstetter, J. Balajka, I. Sokolović, M. Valtiner, F. Mittendorfer, M. Schmid, M. Setvín and U. Diebold, *Nat. Commun.*, 2023, **14**, 208.
